# In Vitro Antischistosomal Activity of *Bridelia ferruginea*, *Clausena anisata*, *Khaya senegalensis*, and *Vernonia amygdalina*

**DOI:** 10.1155/2024/8074291

**Published:** 2024-03-21

**Authors:** Deryl Nii Okantey Kuevi, Jennifer Keiser, Cécile Häberli, Abena Konadu Owusu-Senyah, Mawutor Kwame Ahiabu

**Affiliations:** ^1^Council for Scientific and Industrial Research (CSIR), Water Research Institute, Biomedical and Public Health Research Unit, P.O. Box AH 38, Accra, Ghana; ^2^Department of Medical Parasitology and Infection Biology, Swiss Tropical and Public Health Institute, Basel, Switzerland; ^3^University of Basel, Basel, Switzerland

## Abstract

**Background:**

Schistosomiasis is caused by parasitic flatworms and the disease is endemic to most countries in sub-Saharan Africa including Ghana. The current therapeutic agent for managing this disease solely relies on praziquantel. The continual dependence on this single available drug could lead to possible drug resistance. This study seeks to evaluate the antischistosomal activity of the following Ghanaian medicinal plants: *Khaya senegalensis*, *Vernonia amygdalina*, *Clausena anisata*, and *Bridelia ferruginea. Methodology*. Two concentrations (100 *μ*g/mL and 50 *μ*g/mL) of each extract were tested in a 96-well plate containing 30 newly transformed schistosomula (NTS). Moreover, six worms of both sexes of adult *Schistosoma mansoni* were exposed to the extracts diluted in the RPMI medium. The assay was performed in a 24-well plate. The parasitic worms were examined using an inverted optical microscope.

**Results:**

At 100 *μ*g/mL and 50 *μ*g/mL, all extracts performed better and showed strong activity (*p* < 0.001) against NTS; thus, 98.08%, 100%, 80.77%, and 100% for Clausena, Vernonia, Bridelia, and Khaya, respectively, when compared to praziquantel. Strong activity was recorded when the extracts underwent testing against *Schistosoma mansoni* adults at 100 *μ*g/mL; 96.35%, 100%, and 94.55% for Vernonia, Bridelia, and Khaya, respectively, except for Clausena which exhibited weak activity, i.e., 56.02%. There was no significant difference between Vernonia, Bridelia, and Khaya when compared to praziquantel.

**Conclusion:**

At 100 *μ*g/mL, *Khaya senegalensis*, *Vernonia amygdalina*, and *Bridelia ferruginea* extracts demonstrated strong activity against both schistosomula and adult *Schistosoma mansoni*. These data can serve as baseline information in the quest to find alternative therapeutic agents to treat schistosomiasis.

## 1. Introduction

Schistosomiasis continues to be a major health concern, affecting almost 240 million people worldwide particularly in the tropical and subtropical regions, and there are 700 million individuals living in endemic communities [1]. This tropical disease is caused by *Schistosoma* trematodes [2, 3], and it is predominant in poverty-stricken communities with poor sanitation and without potable water [1]. Schistosomiasis in Ghana is mostly caused by *Schistosoma haematobium* and *Schistosoma mansoni* [4, 5]. The lifecycle begins with an infected person shedding eggs through urine or faeces; the eggs hatch and release miracidia under a controlled condition which further penetrates the specific snail intermediary host. Sporocysts and cercariae are developed in the snail due to the free-swimming nature of the cercariae, and it is released into water which later penetrates the skin of man. The cercariae loses its tail during penetration and develops into a schistosomulum which goes into circulation. The schistosomula resides in the liver and matures into adult worms. The adult worms pair up to reside in the bowel/rectum or bladder and start laying eggs which later shed in stool or urine.

Currently, the main therapeutic agent for the management of schistosomiasis is praziquantel [6, 7]. The drug is widely used for mass drug administration in schistosomiasis endemic areas [8]. It is projected that 57.4 million children in Africa received treatment for schistosomiasis in 2017, representing a coverage of 57.2% based on the World Health Organization's weekly epidemiological reports from 2017. Just 9.6 million adults were said to have received treatment, translating to a 10.9% coverage rate [9]. Ghana's population with schistosomiasis increased from 10.1 to 11.5 million between 2017 and 2021; however, only 24.1% of those in need of preventive treatment received it [10]. Praziquantel acts by rapidly increasing the concentration of calcium (Ca^2+^) influx within the schistosome, which causes the worms' muscles to contract and become paralyzed [11]. Other early effects of praziquantel include morphological changes like darkening and damage to the mature worm's tegumental surface [12]. The schistosome antigens at the parasite surface become more visible owing to these morphological changes [4]. The worms are either destroyed in the intestine or transferred into the stool [13].

Remarkably, praziquantel is not very effective against young schistosomes [14, 15]. Despite its efficacy against this neglected tropical disease, its unpleasant taste coupled with several side effects have resulted in noncompliance with the treatment regimen [16]. Adverse events include stomach pain, sickness, and emesis [2]. Owing to the continual dependence on this single available drug could lead to possible drug resistance [17]. Therefore, it is prudent to conduct intensive research to find leads with pharmacological efficacy and safety data to fight this parasitic disease. This initiative supports the WHO strategy to eliminate schistosomiasis by 2030 [1].

Natural products are potentially valuable sources of new leads. Natural products and their derivatives have contributed to about 25–30% of drugs on the market currently [18]. Approximately 80% of the developing countries with about sixty percent of people worldwide use traditional herbal therapy to manage or treat their illnesses [19]. Ghana has a rich plant biodiversity and for years, various herbal preparations have been used to treat afflictions and diseases such as malaria, cholera, helminth infection, typhoid fever, osteoarthritis, stomach ulcer, male infertility, and anaemia [20]. Schistosomiasis is no exception as previous works have proved the therapeutic nature of some medicinal plants against the adult worm [3–5, 12].

*Clausena anisata* is an evergreen shrub in the Rutaceae family that grows abundantly throughout Africa [21]. It has been determined that the plant's many morphological components work well as treatments for helminthiasis, respiratory issues, cardiac problems, hypertension, malaria fever, rheumatism, insanity, convulsions, and other inflammatory illnesses [22].

*Bridelia ferruginea* from the Euphorbiaceae family is a widespread type of shrub in the moister regions [23]. According to Olajide, the plant is used to cure burns, boils, dislocations, and burns in addition to treating arthritis. Fever, headaches, stiffness, and rheumatic symptoms are treated with pulped bark tea, which is also applied locally to cure oedemas [24].

*Vernonia amygdalina* from the Asteraceae family is a tiny plant mostly found in tropical Africa but has been domesticated in various regions of West Africa. It has rough bark and dark green foliage [25]. It is now widely used for managing and treating several disorders. The leaves are useful components in the making of herbal remedies. The many isolated biologically active substances are responsible for the wide range of physiological effects. The effectiveness of these metabolites against parasites, especially worms, has been well documented. The modes of action include worm paralysis, interference with energy production, and impairments of nutrition uptake, motility, and reproduction. [25]. Vernonia extract has been studied for its molluscicidal properties against the mollusc intermediary host of schistosoma and other pathogenic worms as a method of schistosomiasis control. According to reports, adult *Biomphalaria pfeifferi* is toxic to the plant's hydrophilic extracts [26].

*Khaya senegalensis* belongs to the Meliaceae family of trees. It is a deciduous tree with an 8–16 m clean bole with buttresses that are neither noticeable nor present, growing 15–30 m high and up to 1 m in diameter; flowers are dark pink to bright crimson, producing a red resin; and bark dark grey, with tiny, thin, and reddish-tinged scales [27]. It has a wide range of therapeutic uses, including antimalarial and antibacterial properties. *Plasmodium falciparum* toxicity of the stem bark extract has already been demonstrated. It is also apparent in literature that the stem bark of *Khaya senegalensis* possesses antifungal, antiprotozoal, anthelmintic, and anticancer effects, as well as free radical scavenger activities [28].

This study seeks to assess the in vitro antischistosomal activity of important Ghanaian medicinal plants, namely, *Khaya senegalensis*, *Vernonia amygdalina*, *Clausena anisata*, and *Bridelia ferruginea.*

## 2. Methods

### 2.1. Collection and Preparation of Plant Materials

Stem barks of *Khaya senegalensis* and leaves of *Vernonia amygdalina*, *Clausena anisata*, and *Bridelia ferruginea* were collected and authenticated at the Centre for plant medicine research, Mampong, Ghana. The herbarium contained a specimen serving as a voucher for the plant materials. The collected stem bark and leaves of the various plants were cleaned to remove debris, air dried, and pulverized into powdered form. The pulverized samples were stored in sealed containers prior to extraction.

### 2.2. Preparation of Plant Extracts

The maceration technique was employed in extracting the plant materials. The powdered plant samples (500 g) were poured into conical flasks containing ethanol (1.5 L) for extraction at room temperature (28°C). Plant samples were fully submerged in the solvent with the conical flask stoppered. The mixture was allowed to stand for 72 hours with intermittent agitation. The mixture was then filtered with cotton first and then refiltered with filter paper. At 40°C, the filtrate was concentrated under decreased pressure in a rotary evaporator. The slurry extract from the rotary evaporator was dried using the oven at 40°C. The dried extract was stored in a desiccator awaiting the antischistosomal assay.

### 2.3. Phytochemical Screening of Plant Extracts

To identify the type of phytoconstituents and their presence in plant parts, preliminary phytochemical analysis was done using several chemical reagents. Standard qualitative techniques were used to assess the presence of steroids, flavonoids, tannins, alkaloids, saponins, glycosides, and phenolics [29].

### 2.4. Attenuated Total Reflection-Fourier Transform Infrared (ATR-FTIR) Spectroscopy Analysis of Plant Extracts

ATR-FTIR analysis of the plant extracts was performed using Attenuated Total Reflection Fourier Transform Infrared spectrometer. Plant extracts were placed on the crystal and the sample was scanned across a wave number range to perform the analysis of 4000 to 400 cm^−1^.

### 2.5. *Schistosoma mansoni* Adult Worms and Schistosomula

The Swiss Tropical and Public Health (Swiss TPH) Institute internally maintains the *Schistosoma mansoni* (Liberian strain) life cycle. The collected cercariae were manually turned into schistosomula employing a method that was modified [30]. NTS were incubated for 12–24 hours at 37°C with 5% CO_2_. Adult Schistosoma mansoni of both sexes were obtained and placed in Roswell Park Memorial Institute (RPMI) media, supplemented with fetal calf serum (FCS) and penicillin-streptomycin.

### 2.6. In Vitro Assays

In assaying the freshly converted schistosomula, the solution containing the parasite was measured to 30–40 NTS/50 *μ*L in enriched M199 medium and administered to the extract dilutions in 96-well plates. The newly transformed schistosomula were previously exposed to extract of 100 *μ*g/mL (0.1% dimethyl sulfoxide) followed by 50 *μ*g/mL. The testing of adult worms followed the identification of hit compounds on newly transformed schistosomula. Males, females, and pairs (6 worms of both sexes (three each) per well) were exposed to the extract diluted in the added RPMI medium. The assays were performed in 24-wells plates (Eppendorf AG, Hamburg, Germany). For extracts with high activity against NTS and adults (effect 75% at 100 g/mL and 50 g/mL after 72 hours), mortality effects in percentages were calculated. Each assay on NTS was performed in triplicate and repeated once; the adult assays were conducted in duplicates. Negative controls with the highest dimethyl sulfoxide (DMSO) concentration and praziquantel as the positive control were used for both NTS and adult worms. Three days after the extract was exposed, and the parasitic worms were examined using an inverted optical microscope [30, 31].

### 2.7. Statistical Analysis

All data were expressed as the mean ± standard deviation (SD). Percent mortality bar graphs were created for the in vitro antischistosomal activities, respectively. GraphPad Prism for Windows version 7 (GraphPad Software, San Diego, CA, USA) was used for all statistical analyses. *p* < 0.05 was considered statistically significant.

## 3. Results and Discussion

### 3.1. Phytochemical Analysis

The screening of phytochemicals was done to qualitatively determine the presence of certain phytoconstituents present in the leaves of *Vernonia amygdalina*, *Bridelia ferruginea*, and *Clausena anisata* and the stem bark of *Khaya senegalensis*. The phytochemical components of medicinal plants are regarded to be responsible for their pharmacological effectiveness. According to Tables 1 and 2, the results showed that all four extracts had alkaloids, steroids, glycosides, tannins, flavonoids, and saponins with the exception of *Bridelia ferruginea* where steroids was not detected. From the quantitative analysis, phenols were the most abundant in all the extracts. Similar studies reported by other researchers showed that *Khaya senegalensis* contained saponins, flavonoids, tannins, glycosides, and steroids [32, 33]. Muraina et al. [34] and Oyeyemi et al. [25] reported the presence of alkaloids, saponins, flavonoids, phenolic acids, and steroids in *Vernonia amygdalina* [25, 34]. This corroborates the findings in this study. According to Makut and colleagues, alkaloids, saponins, and tannins are the common phytochemicals known to cause parasitic paralysis or death [32]. All four extracts eliciting antischistosomal activity could be attributed to the presence of either alkaloids, saponins, or tannins working synergistically or addictively acting at single or multiple target sites [35].

### 3.2. ATR-FTIR Analysis of Extracts

**Figure 1:**
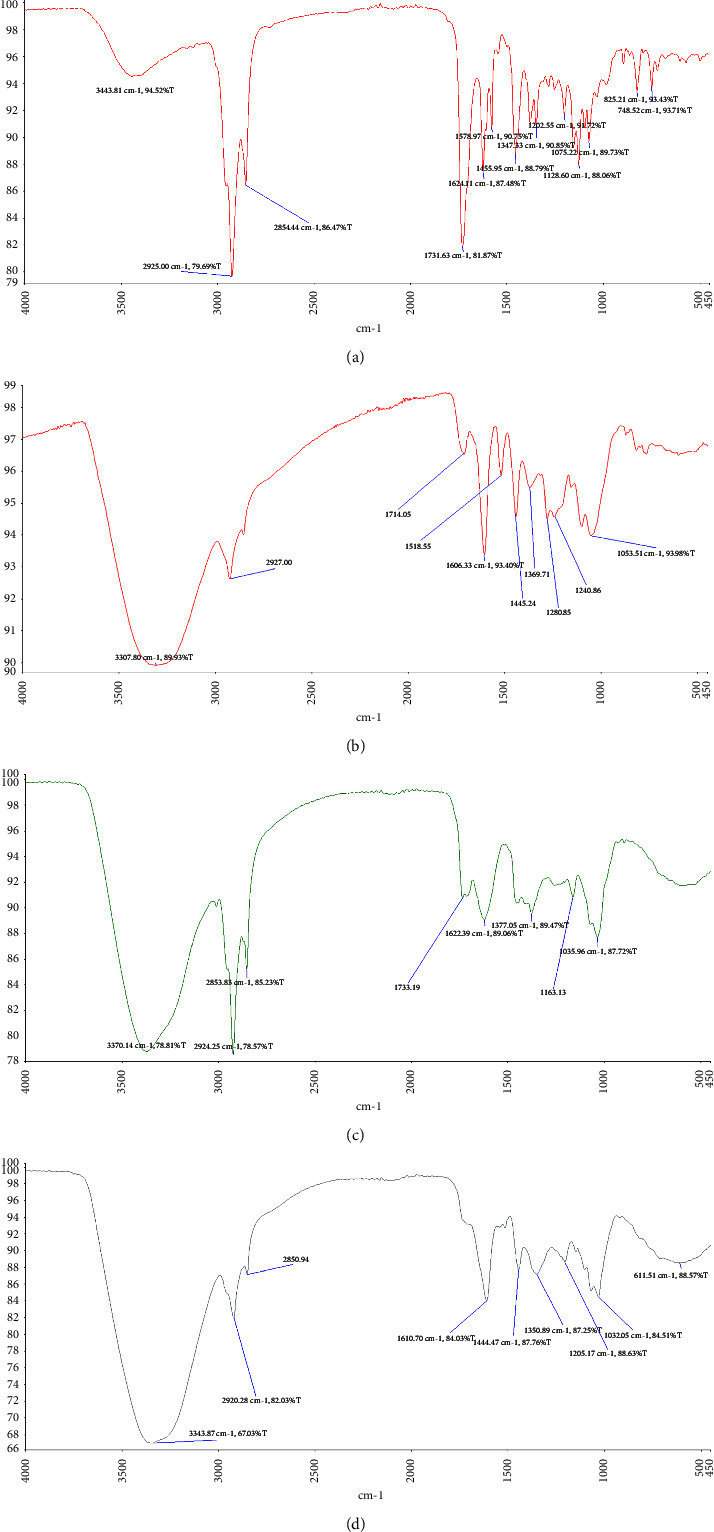
FTIR spectra of ethanolic leaf extracts of *Clausena anisata* (a), *Khaya senegalensis* (b), *Vernonia amygdalina* (c), and *Bridelia ferruginea* (d).

The IR spectrum of the different extracts revealed structural information about phytochemical constituents [35]. The FTIR data showed the presence of hydroxyl, carbonyl, and carboxylic functional groups in all the extracts. Broad peaks at 3443 cm^−1^, 3370 cm^−1^, 3343 cm^−1^, and 3307 cm^−1^ were recorded for *Clausena anisata*, *Vernonia amygdalina*, *Bridelia ferruginea*, and *Khaya senegalensis*, respectively (Figure 1). These broad peaks are due to the O–H stretching hydroxyl group which is indicative of polyhydroxy compounds consisting of flavonoids and saponins which attests to the findings of the phytochemical analysis. Peaks at 1731 cm^−1^, 1622 cm^−1^, 1610 cm^−1^, and 1606 cm^−1^ for *Clausena anisata*, *Vernonia amygdalina*, *Bridelia ferruginea,* and *Khaya senegalensis*, respectively, indicate the presence of C=O stretch of a carbonyl and carboxylic functional group. This corresponds to the presence of saponins in the extracts.

### 3.3. Antischistosomal Activity of Extracts against NTS

**Figure 2:**
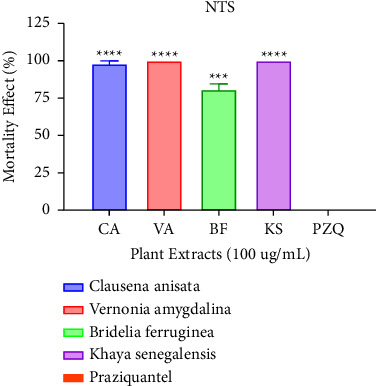
Mortality effect observed on NTS after treating with the plant extracts (100 *μ*g/mL) after 72 hours. Each plant treatment group was compared to praziquantel at *p*=0.05 significant difference.

**Figure 3:**
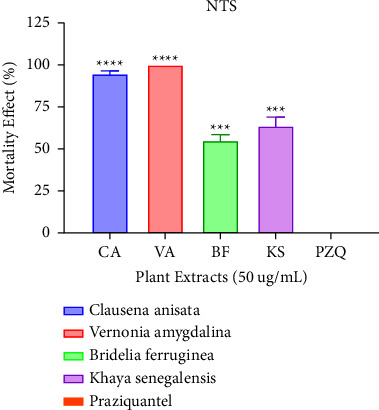
Mortality effect observed on NTS after treating with the plant extracts (50 *μ*g/mL) after 72 hours. Each plant treatment group was compared to praziquantel at *p*=0.05 significant difference.

At a concentration of 100 *μ*g/mL, all four extracts had high activity against NTS. *Khaya senegalensis* (KS) and *Vernonia amygdalina* (VA) extracts recorded the highest percentage activity followed by *Clausena anisata* (CA) and *Bridelia ferruginea* (BF) as shown in Figure 2. There was no recorded mortality for praziquantel since it is less effective against juvenile worms. After administering 100 *μ*g/mL, the concentration of the extracts was halved to 50 *μ*g/mL. From Figure 3, it can be inferred that Vernonia was the most potent having a percentage activity of 100%. This corroborates a study reported by Balahbib and colleagues which revealed that the *Punica granatum* leave extract elicited 100% death rate against schistosomula [3]. Similar results were reported by Chacha and colleagues which showed that *Searsia longipes* and *Lannea schimperi* exhibited 100% mortality against schistosomula after six hours of administration.

### 3.4. Antischistosomal Activity of Extracts against *Schistosoma mansoni*

**Figure 4:**
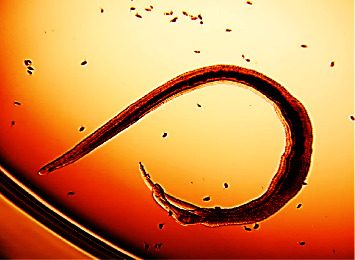
*Schistosoma mansoni* adult worm alive.

**Figure 5:**
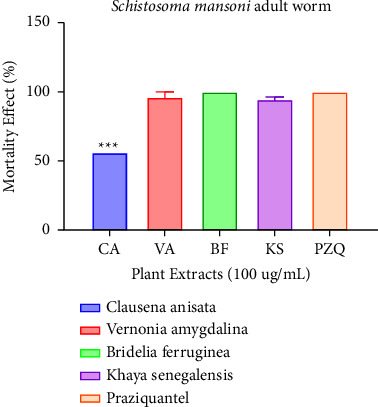
Mortality effect observed on *Schistosoma mansoni* adult worm after treating with the plant extracts (100 *μ*g/mL) after 72 hours. Each plant treatment group was compared to praziquantel at *p*=0.05 significant difference.

**Figure 6:**
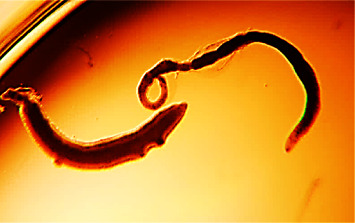
Dead *Schistosoma mansoni* adult worm after treating with plant extracts.

The activity of all four plant extracts were tested against *Schistosoma mansoni* adult worms and was compared to praziquantel as shown in Figure 4. A concentration of 100 µg/mL of all the four extracts was used and from Figure 5, it can be inferred that *Bridelia ferruginea* exhibited the highest activity with a percentage mortality effect of 100 followed by *Vernonia amygdalina* and *Khaya senegalensis*. There was no significant difference when Bridelia, Khaya, and Vernonia were compared to praziquantel. It is worthy to note that the palsy and/or mortality observed in Figure 6 might be linked to secondary metabolites such as alkaloids, saponins, and tannins found in the ethanolic extracts of *Bridelia ferruginea*, *Vernonia amygdalina*, *and Khaya senegalensis*. The worms' motility may have been inhibited, they may have been paralyzed, or they may have died as a result of these plant metabolites acting alone or in combination. A study done by Acheampong and colleagues showed that Vernonia was effective against *Schistosoma mansoni* in murine models [5]. Interestingly, literature reports have shown that Vernonia has relatively higher activity against helminths as compared to orthodox drugs such as ivermectin and albendazole [36]. Purportedly, the mechanism of action of Vernonia is possibly preventing adult worms from mating and/or preventing female worms from producing eggs [5]. Similar results were reported by Fabri and colleagues when 100 *μ*g/mL of *Mitracarpus frigidus* extract caused complete paralysis of the *Schistosoma mansoni* and complete darkening of the tegument [37]. In addition, a study done by De Oliveira showed that 130 µg/mL of *Baccharis trimera* extract rendered *Schistosoma mansoni* adult worms dead followed by morphological alterations on the tegument [38].

**Figure 7:**
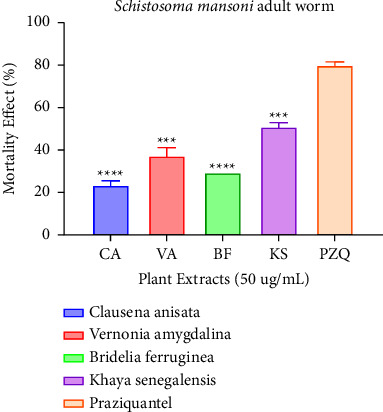
Mortality effect observed on *Schistosoma mansoni* adult worm after treating with the plant extracts (50 *μ*g/mL) after 72 hours. Each plant treatment group was compared to praziquantel at *p*=0.05 significant difference.

The concentration of the four plant extracts was reduced to 50 *μ*g/mL and then tested against *Schistosoma mansoni*. None of the extracts showed significant activity as shown in Figure 7. This could be attributed to the fact that perhaps most of the phytochemicals were in low quantities at that concentration. Phytoconstituents such as tannins, alkaloids, and saponins have been linked to anthelmintic activity [39, 40].

## 4. Conclusion

*Khaya senegalensis*, *Vernonia amygdalina*, and *Bridelia ferruginea* extracts exhibited strong activity against both schistosomula and adult *Schistosoma mansoni* worm at 100 µg/mL, which is very encouraging. Given the medicinal potential of these plant extracts, more research is required to determine the molecular pathways and identify the precise compounds causing the antischistosomal activity [41–43].

## Figures and Tables

**Table 1:** Phytochemical screening of plant extracts.

Phytochemicals	*Clausena anisata*	*Vernonia amygdalina*	*Bridelia ferruginea*	*Khaya senegalensis*
Alkaloids	+	+	+	+
Flavonoids	+	+	+	+
Glycosides	+	+	+	+
Phenolics	+	+	+	+
Saponins	+	+	+	+
Steroids	+	+	—	+
Tannins	+	+	+	+

+: detected, —: not detected.

**Table 2:** Quantitative phytochemical analysis of plant extracts.

Phytochemicals	*Bridelia ferruginea*	*Clausena anisata*	*Khaya senegalensis*	*Vernonia amygdalina*
Alkaloids (%)	3.14	3.18	3.34	3.74
Flavanoids (%)	3.74	3.34	4.14	4.37
Glycosides (%)	3.37	3.24	4.74	4.87
Phenolics (mg/L)	8.17	4.74	9.37	10.43
Saponins (%)	4.14	3.37	5.14	5.74
Steroids (%)	0.00	2.34	3.18	3.14
Tannins (%)	4.74	3.14	5.14	5.74

## Data Availability

The data used to support the findings of this study are available from the corresponding author upon request.
